# Congo red fluorescence enhances digital pathology workflow in cardiac amyloidosis

**DOI:** 10.1038/s41598-025-07157-5

**Published:** 2025-07-11

**Authors:** Giorgio Cazzaniga, Monica De Gaspari, Vincenzo L’Imperio, Carlo Beretta, Angela Greco, Stefania Rizzo, Cristina Basso, Fabio Pagni

**Affiliations:** 1 Pathology, IRCCS Fondazione San Gerardo dei Tintori, Monza, Italy; 2 Department of Medicine and Surgery, University of Milano-Bicocca, Monza, Italy; 3 Cardiovascular Pathology Unit, Azienda Ospedaliera, Department of Cardiac, Thoracic, and Vascular Sciences and Public Health, University of Padua, Padua, Italy; 4 Department of Pathology, ASST Papa Giovanni XXIII, Bergamo, Italy; 5 Department of Medicine and Surgery, Pathology IRCCS Fondazione San Gerardo dei Tintori, University of Milano-Bicocca, Monza, Italy

**Keywords:** Digital pathology, Cardiac amyloidosis, Artificial intelligence, Congo red, Cardiovascular pathology, Computational biology and bioinformatics, Cardiology

## Abstract

Despite advances in non-invasive methods, endomyocardial biopsy (EMB) remains essential for definitive diagnosis of amyloidosis in many cases. Traditionally, Congo red birefringence (CRB) has been crucial for identifying amyloid deposits but is challenging to capture digitally. Emerging fluorescent Congo red imaging (CRF) overcomes this problem and holds promise in image analysis and AI applications. The diagnostic performance of CRF on virtual slides was evaluated in a cohort of EMB and autopsy cases. The feasibility of developing AI algorithms applicable to centers lacking a fluorescence scanner was investigated leveraging a computational pipeline that enables fluorescence outcome visualization in brightfield. The study analyzed 43 digital myocardial slides stained with Congo Red, acquired using a fluorescent Texas Red filter. Among these, 28 (65%) were diagnosed with amyloidosis, with complete diagnostic agreement with original diagnosis. AI achieved an AUC-ROC of 0.87, 0.86 and 0.79 on the training, validation and test set, respectively, in tile-level classification for amyloidosis positivity and IoU and Dice scores indicating partial but reasonable overlap between predictions and ground truth in amyloid segmentation. The study underscores CRF’s transformative impact on virtual slides and AI integration for diagnosing cardiac amyloidosis, showcasing high reliability and diagnostic accuracy. These advancements promise a more quantitative and precise approach, facilitating the histological study of the disease in the digital transition era of pathology labs.

**Supplementary Information:**

The online version contains supplementary material available at 10.1038/s41598-025-07157-5.

## Introduction

Cardiac amyloidosis (CA) is caused by the extracellular deposition of fibrils composed of low molecular weight subunits from a wide range of precursor peptides/proteins^1,2^, leading to progressive cardiac dysfunction^3^. The majority of CA cases are due to the deposition of fibrils composed of monoclonal immunoglobulin light chains (AL) or transthyretin (TTR). Early diagnosis is crucial, as disease-modifying treatment slowing or halting disease progressions is available, and survival is mostly determined by the severity of heart involvement^4^. The diagnostic algorithm includes both invasive and non-invasive criteria to reach the diagnosis. Depending on the clinical scenario, an endomyocardial biopsy (EMB) can be required to obtain the histological confirmation of amyloid deposits in the heart.

The recent introduction of digital and computational pathology has impacted also the field of cardiac pathology^5,6^. Congo red (CR) birefringence (CRB) is still routinely applied in the histological diagnosis of CA and is considered pivotal for deposits detection in the field of renal pathology^7^. Although scanners capable of digitizing birefringence do exist, their limited distribution due to high costs and a focus on research applications hinder widespread adoption. Moreover, the difficulty of detecting the diagnostic CRB of the dye can cause a low sensitivity^8,9^. An encouraging approach lies in harnessing the fluorescent properties of CR when bound to amyloid, a characteristic that can be utilized by increasingly available darkfield-specific or hybrid scanners to make the transition to digital slides available and leverage the advantages of image analysis and AI^10^. To further expand the wider implementation of AI tools in pathology the new digital workflows can benefit from practical, democratic, and easily integrable solutions. In this sense, leveraging Congo red fluorescence represents a more accessible approach, albeit still underexplored, compared to second-level techniques such as infrared microscopy and omics technologies^11,12^. The aim of the present study is to assess the diagnostic performance of CR fluorescence (CRF) on virtual slides compared to the routine diagnostic practice in a cardiovascular pathology center, and to apply image analysis techniques (Streamlined Pipeline for Amyloid Detection Through Congo Red Fluorescence Digital Analysis, SPADA) in order to screen whole slides cases for small deposits of amyloid.

## Methods

### Cases

A consecutive series of CA cases including both EMB and myocardial autopsy specimens was retrospectively retrieved at the Cardiovascular Pathology Unit of Padua University, Italy (from January 2018 to December 2023). The original diagnosis of CA was made at histology with a set of stainings including Hematoxylin-Eosin, CR, Thioflavin T (fluorescence), and Sulfated Alcian Blue (SAB). Available clinical information was collected (sex, age, amyloid type, diagnosis of CA before death and whether amyloid was the primary cause of death for autopsy cases). Control cases were selected with an original diagnosis negative for CA, either on EMB or at whole heart examination from autopsy. Approval was obtained from the PNRR-MR1-2022-12375735, 03/16/23 (Fondazione IRCCS San Gerardo dei Tintori) and from the 280n/AO/22 (Azienda Ospedaliera di Padova) ethics committee. The study was conducted in compliance with the Declaration of Helsinki. All data were fully anonymized and informed consent was waived by the Fondazione IRCCS San Gerardo dei Tintori (PNRR-MR1-2022-12375735) and Azienda Ospedaliera di Padova (280n/AO/22) ethics committee in accordance with applicable ethical guidelines.

### Digital pathology and automated image analysis pipeline

Whole slide images (WSIs) were acquired using NanoZoomer S60 (Hamamatsu, Shizuoka, Japan) at 20× magnification in brightfield and darkfield mode using two fluorescence filter combinations, as previously described^7^:

- Texas red filter—TRITC—(556/20 excitation—617/73 emission).
- A scramble filter combination to detect autofluorescence in tissue (480/17 excitation—617/73 emission).

**Fig. 1:**
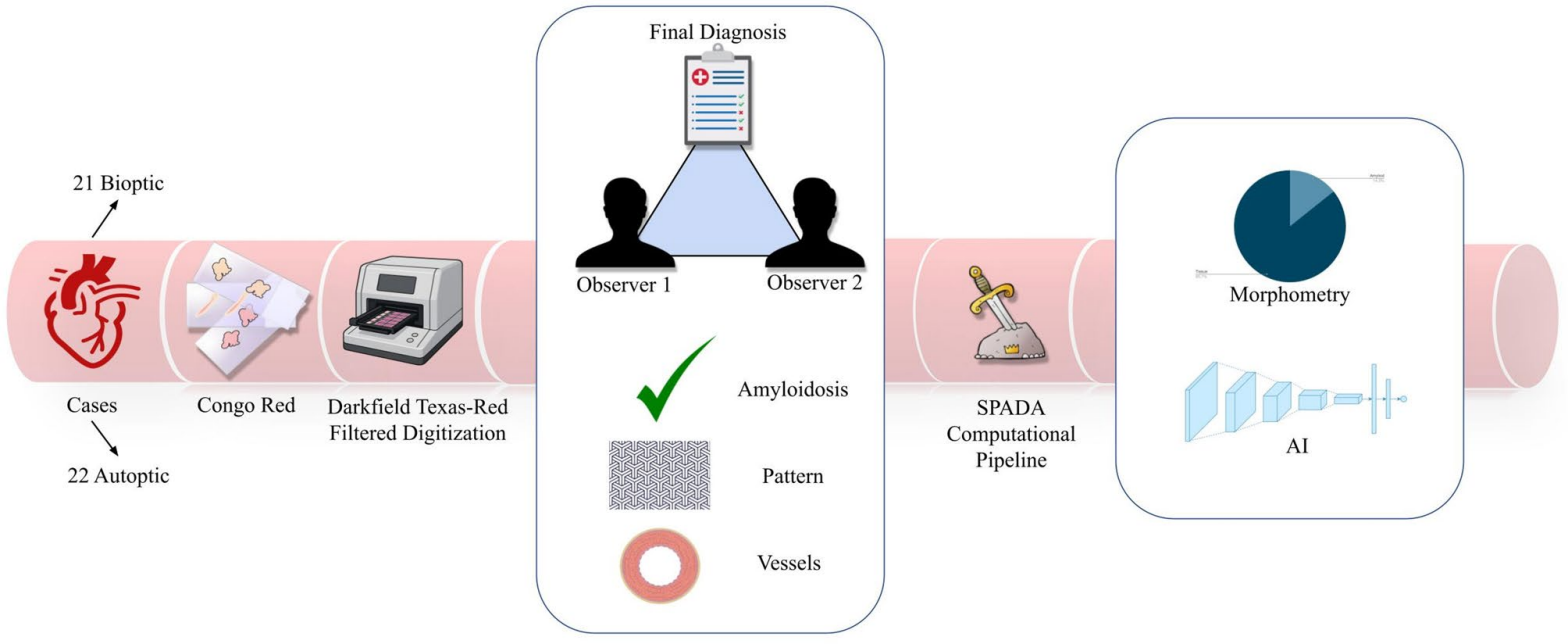
Study design: Congo Red-stained slides were digitized using a Texas Red filter on a fluorescence scanner. Two pathologists assessed amyloid presence, deposition pattern, and vascular involvement, comparing results with the original diagnosis. The computational pipeline SPADA facilitated morphometric analysis and AI model development. The third image from the left was generated by the author using an AI-based image generator (OpenAI DALL·E).

An expert pathologist (FP) and a pathology trainee (CBe) evaluated the CRF on virtual slides blindly to the original diagnosis (presence/absence of amyloid deposits); in case of positive detection, the distribution pattern (diffuse pericellular, discrete pericellular, or nodular), and the presence of vascular involvement (in terms of absence, presence with or without obstruction) were recorded. Then, the SPADA pipeline was applied on CRFs to automatically segment amyloid deposits and extract quantitative data including quantitative amyloid percentage (defined as amyloid burden and calculated as area covered by amyloid deposits / whole tissue area)^7^. (Fig. 1) The SPADA pipeline utilizes WSI scanning with a Texas Red filter, enhancing red frequencies predominantly expressed by amyloid, and tissue autofluorescence scanning. This creates a third WSI by subtracting the two, yielding an image containing only amyloid deposits in binary form, which can easily be used to transfer annotations to the brightfield image without the need for co-registration of slides from the same specimen. The results obtained from the CRFs were then compared between the two pathologists and, after resolving discrepancies by consensus, with the original diagnoses of the EMB or myocardial samples from autopsy to determine the concordance and reliability of the assessments.

### AI for cardiac amyloidosis

After transferring the annotations obtained with SPADA onto the brightfield CR virtual slides, tiles of 256 × 256 pixels were extracted, in their original format and with a dark background outside the SPADA-derived amyloid annotation (binary mask), at 20X magnification (0.4415 μm/px), corresponding to a square of about 113 × 113 μm of tissue. A custom script, specifically designed to be executed in QuPath^13^, was developed to generate both the tiles and the respective masks containing the ground truth tissue annotations, after tissue detection. Tiling was subjected to 10-fold nested cross-validation to ensure a robust evaluation of the model’s performance and mitigate overfitting. This approach involves iteratively training the model on different subsets of the data, with each fold being split at the case level into 80% for training, 10% for validation, and 10% for testing. The model was trained on different data subsets while using separate validation and test sets for each fold, providing a more reliable estimate of generalizability and model robustness across varying data splits.During the training phase, color augmentation was employed to address the high variability present in this type of staining, even within the same laboratory, aiming to enhance the model’s generalizability beyond traditional augmentation methods.

Two separate experiments were conducted to investigate the capabilities of AI in detecting and recognizing amyloid deposits:

- The first experiment focused on classifying tiles, specifically to determine whether they contained amyloid deposits. Tiles extracted from the brightfield CR virtual slides were labeled as either positive or negative for amyloidosis based on the SPADA-derived annotations. A convolutional neural network (CNN) classifier, specifically an EfficientNet model, was then trained to perform this binary classification task (presence vs. absence of deposits). The classifier’s performance was evaluated using accuracy, F1-score and AUC-ROC. To evaluate model performance at the WSI level, tile-level predictions were first aggregated for each slide. Specifically, the average probability across all tiles belonging to a given slide was computed, yielding a single slide-level probability score. A threshold of 0.5 was then applied to this aggregated score to assign a binary class label (positive or negative) to the entire slide. Predicted slide-level labels were compared to the corresponding ground truth annotations to determine the number of true positives (TP), true negatives (TN), false positives (FP), and false negatives (FN).
- In the second experiment, a Segformer segmentation model was trained to automatically segment amyloid-containing areas directly on the brightfield CR slides, without the need for CRB or CRF. The performance of the model was assessed using the Intersection over Union (IoU) and Dice Coefficient. These metrics quantified the similarity and diversity of the predicted amyloid areas against the ground truth annotations and the overlap between the predicted and ground truth amyloid regions, emphasizing both precision and recall by considering twice the intersection over the sum of predicted and actual areas, respectively.

We investigated the interpretability of a deep learning model trained for image classification using heatmap overlays based on Gradient-weighted Class Activation Mapping (Grad-CAM). The objective was to identify which regions of the images the model predominantly relied upon for its predictions. Both experiments were conducted on a workstation equipped with an Intel Core i7 processor, 16 GB of RAM memory, and a NVIDIA GeForce GTX 1070 Max-Q graphics card.

### Statistical analysis

For continuous variables, mean and standard deviation (SD) were calculated, while qualitative variables were reported as count and frequency. For the comparison of means and qualitative variables, the t-test, fisher’s exact test and χ2 test were used, considering P values < 0.05 as statistically significant. The effect size was calculated using Cohen’s d to assess the magnitude of the difference between groups. Concordance rate, sensitivity, specificity, positive and negative predictive values, and Cohen’s kappa coefficient (κ) were calculated to evaluate the inter-observer variability of the two pathologists and their agreement with the original diagnoses. Spearman correlation test was used to compare the amyloid burden between different methods. Effect size evaluations were conducted using Cohen’s d for continuous variables to assess the magnitude of differences observed. Statistical analyses were performed using Excel 2016 (Microsoft) and Python 3.10, utilizing the pandas and scikit-learn libraries.

## Results

### Cases

A total of 43 CR slides, including 22 autopsy slides (51%) represented by full-thickness sections of the left ventricular wall and 21 EMB (49%), were evaluated. Of these, 28 (65%) were originally diagnosed with CA, 18 and 10 in the autopsy and EMB group, respectively. Controls included normal myocardium, myocarditis, and chronic ischemic heart disease. The main clinical and pathological features evaluated in CA cases are reported in Table 1.

**Table 1:** Clinical and pathological features of the 28 CA cases with available CR stain, subdivided by amyloid type.

	ATTR (*N* = 17)	AL (*N* = 11)	*P* value
Age	78 (75–82)	69 (56.2–72.8)	< 0.01
Sex, F	3 (17.6)	3 (27.8)	0.54
EF	47.0 (29.0-56.7)	43.0 (29.7–57.3)	0.97
Type, EMB autopsy	7 (41.2) 10 (58.8)	6 (54.5) 5 (45.5)	0.49
In vivo diagnosis (autopsy-only)	6/10 (60)	4/5 (80)	0.44
Primary cause of death amyloid (autopsy-only)	7/10 (70)	5/5 (100)	0.17
Pattern, original evaluation Pericellular discrete Pericellular diffuse Nodular	2 (11.8) 4 (23.5) 11 (64.7)	8 (72.7) 2 (18.2) 1 (9.1)	< 0.01
Vascular involvement, original evaluation Absent Present, non-obstructive Present, obstructive Not assessable	14 (82.4) 2 (11.8) 1 (5.9) 0 (0)	3 (27.8) 3 (27.8) 4 (36.4) 1 (9.1)	0.03
Amyloid burden, original evaluation	32.0 (10.7–61.0)	26.0 (17.2–44.7)	0.57

Data are expressed as n (%) or as median (IQR).

*ATTR* transthyretin amyloidosis,* AL* immunoglobulin light chain amyloidosis,* F* female,* EF* ejection fraction,* EMB *endomyocardial biopsy.

### Diagnostic performance and inter-observer variability of CRFs

**Fig. 2:**
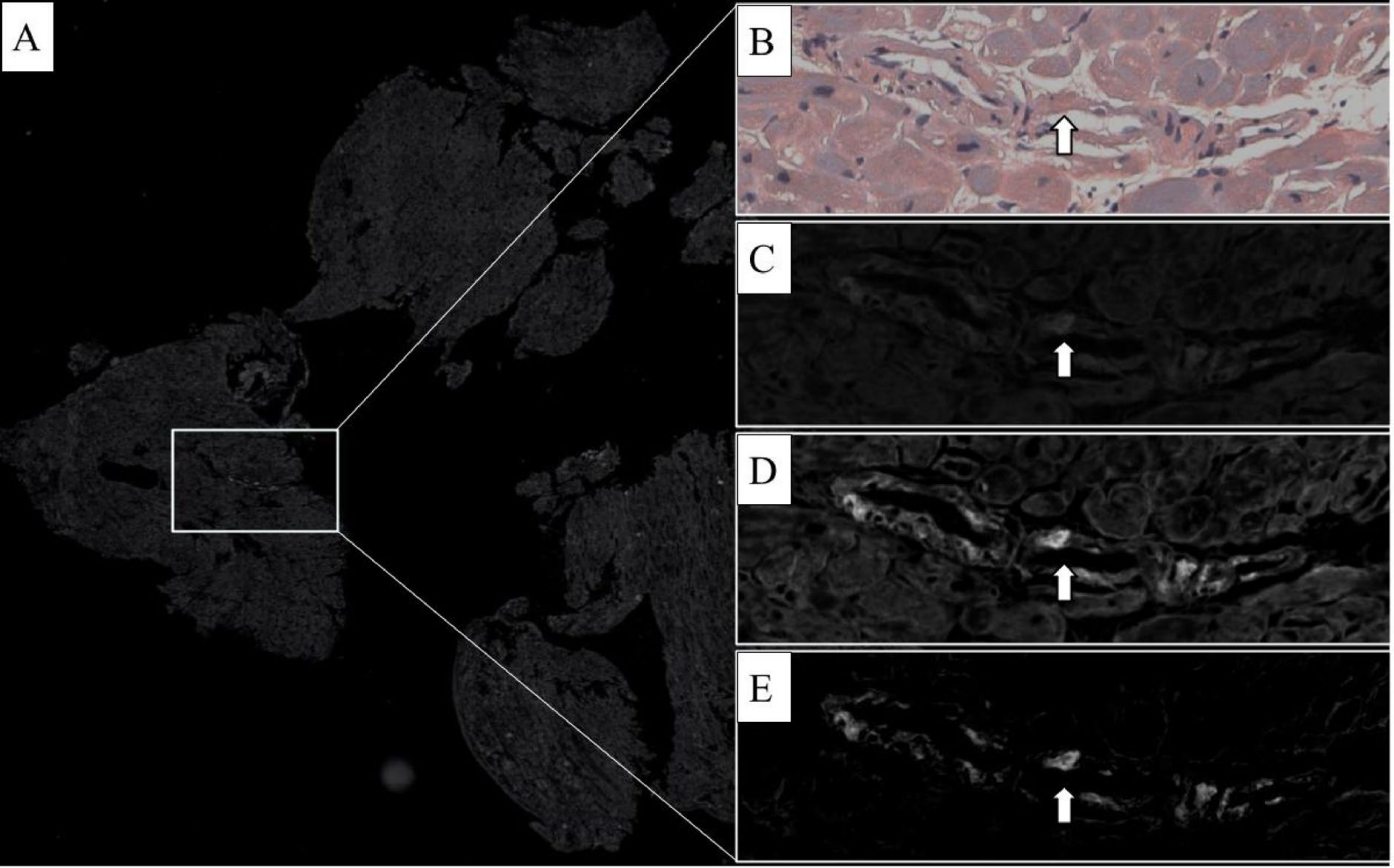
Minimal vascular positivity was initially missed by one of the two pathologists. (**a**) shows a low-magnification view of the fluorescence slide using the Texas Red filter. (**b**) highlights the suspicious area in brightfield, while (**c**) presents the same area under autofluorescence. (**d**) shows the suspicious area in Texas Red, and (**e**) displays the final processed image after applying the SPADA pipeline, which subtracts autofluorescence. This approach eliminated confounders, allowing both pathologists to reach a consensus and confirm the case’s positivity.

Complete concordance on CA detection was noted between the original and the CRFs-based diagnosis. The comparison of blinded evaluation on CRFs by the two reviewers demonstrated excellent interobserver agreement, with a concordance rate of 0.98 and a Cohen’s k of 0.96 (Supplementary Fig. 1). The only discordant case between the two pathologists involved amyloid presence, primarily observed within the vascular wall of a single small artery on the EMB. By employing the SPADA pipeline, we confirmed the vascular amyloid positivity and were able to reconcile our findings with the original diagnosis. Notably, the initial assessment had indicated both vascular and pericellular amyloid deposition, identified through various staining techniques.(Fig. 2).

Minor discrepancies were observed in the comparison of deposition patterns and vascular involvement between the two pathologists, with a Cohen’s k of 1 and 0.92, respectively. Discordances were resolved by consensus after the visualization of the image obtained with the computational pipeline subtracting the autofluorescence to the CRF image.

Although the overall evaluation of vascular involvement showed good concordance, with 16 negative cases (57%), 10 positive cases (36%), and 2 unscorable cases (7%) due to the absence of vascular structures in the EMB, there were 5 cases with differing classifications. The overall agreement, with a kappa value of 0.61, reflects a generally reliable assessment. The CRF evaluation revealed the following pattern distribution: 16 cases (57%) showed pericellular diffuse patterns, 9 cases (32%) exhibited pericellular discrete patterns, and 3 cases (11%) displayed nodular patterns. In contrast, the original diagnosis showed a predominance of nodular patterns (19 cases, 68%), with fewer pericellular diffuse (5 cases, 18%) and discrete (4 cases, 14%) patterns. Although the kappa value of 0.39 indicates fair agreement, these findings highlight differences provided by CRF.

### Image analysis

**Fig. 3:**
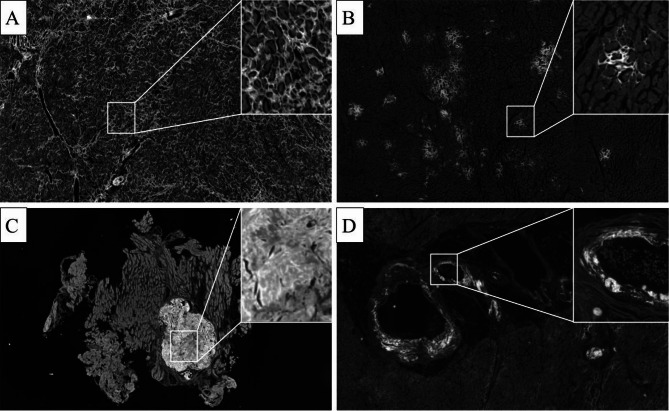
Deposition patterns: (**a**) diffuse pericellular pattern; (**b**) discrete pericellular pattern; (**c**) nodular pattern; (**d**) vascular positivity.

The different patterns of amyloid distribution are illustrated in Fig. 3. The average amyloid substance in the samples was 15% ± 13%, with a minimum deposit of less than 1% and a maximum of 45% in an autopsy sample exhibiting a diffuse pericellular pattern. A strong correlation was observed between the visual assessment of the diffuse pattern and the percentage of amyloid calculated using the SPADA pipeline, with averages of 23 ± 12% for the diffuse pattern and 4 ± 3% for the discrete pattern. In the autopsy cohort, patients who died from amyloidosis had an average amyloid percentage of 15 ± 11%, significantly higher than the 1% average in patients whose primary cause of death was different (*p* < 0.01). No significant differences were found in amyloid levels between the light chain (AL) and transthyretin (ATTR) types (17% ± 15% vs. 13% ± 11%, *p* = 0.546). The comparison with the amyloid burden measured through image analysis on SAB-stained sections shows a significantly higher average value of 31.46% ± 21.14%, compared to the CRF-based image analysis (*p* < 0.001). This difference was statistically confirmed by both the t-test and the Mann-Whitney U test, with an effect size of 0.93. However, a strong positive correlation was found between the two stainings (*r* = 0.72, *p* < 0.001).

### AI

**Fig. 4:**
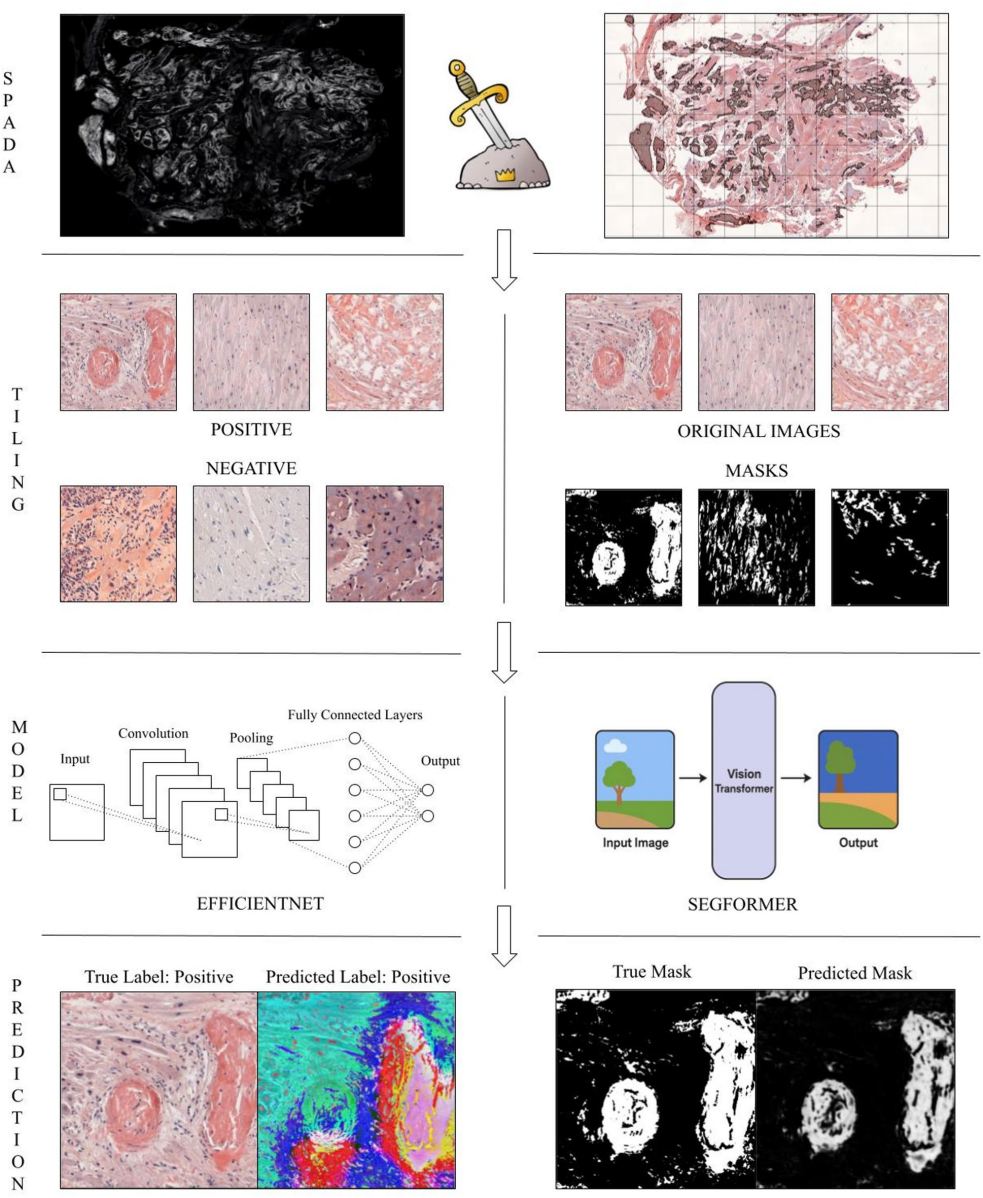
Applications of AI: A mask annotating amyloid deposits was created and overlayed onto the brightfield CR image, enabling extraction of original tiles and their respective masks. In the first experiment (left), positive and negative original tiles were used to train a classifier (based on ResNet18) to recognize the presence or absence of amyloid deposits. Explainability algorithms evaluated areas most influential in the model’s decision-making process. On the right, the segmentation experiment utilized original tiles and corresponding binary masks to train a UNet-based model.

The WSI image processing resulted in the extraction of 28,964 tiles for developing an AI algorithm capable of recognizing amyloid deposits without relying on CRB or CRF(Fig. 4). The model demonstrated great performance across the different datasets, with consistent results in terms of accuracy, F1 score, and AUC-ROC. Notably, the training set yielded the highest metrics (0.87), followed by the validation and test sets, indicating that the model performs well with familiar data but experiences some decrease in generalizability to unseen data. The performance on the test set, in particular, showed a slight reduction (0.79, 0.71 and 0.76, respectively), suggesting room for improvement in its robustness. At the tile level, the model achieved an accuracy of 0.79, sensitivity of 0.89, specificity of 0.68, precision of 0.76, F1 score of 0.82, and an AUC-ROC of approximately 0.79, reflecting a strong capacity to detect amyloid-positive cases, though with some trade-off in specificity.

**Fig. 5:**
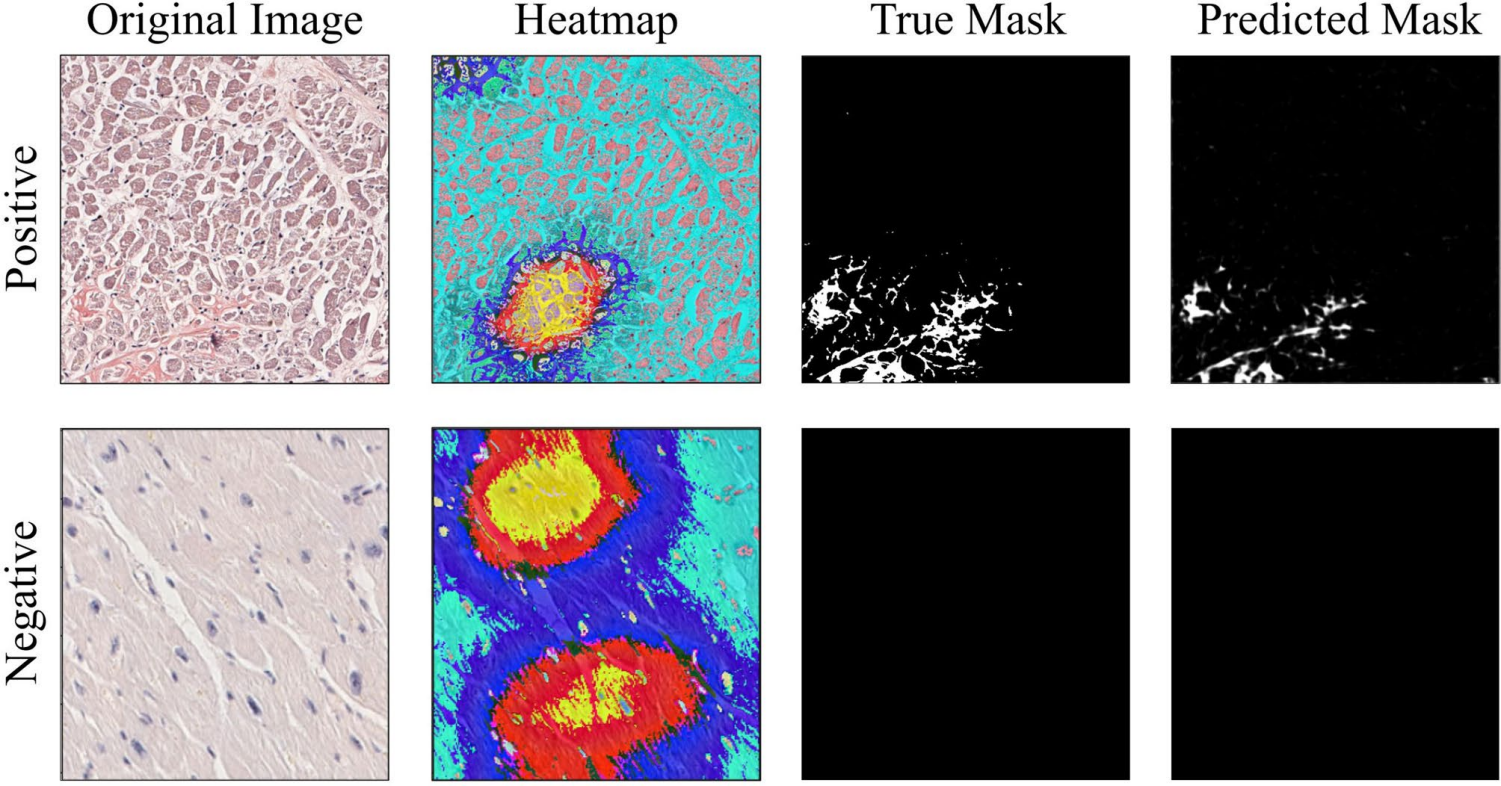
Examples of tile classification with their respective visualizations. From left to right, each set includes: the original image, the classification model’s heatmap highlighting areas most considered (warm colors) and least considered (cool colors) for the decision, the ground truth mask from the segmentation model, and the predicted mask from the segmentation model. In negative cases, the heatmap interpretation consistently highlighted large, nonspecific regions, suggesting a broader area of focus in respect to positive cases where amyloid was pointed out by the heatmap.

By applying a probability threshold of 0.5 at the slide level, the model classified 25 TP, 10 TN, 5 FP, and 3 FN out of 43 whole-slide images. These results closely mirror the metrics observed at the tile level, with an overall accuracy of 0.81, sensitivity of 0.89, specificity of 0.67, precision of 0.83, F1-score of 0.86, and an approximate AUC-ROC of 0.78. These metrics provide a comprehensive evaluation of the model’s classification performance at the slide level, demonstrating its ability to differentiate amyloid-positive from amyloid-negative cases using the chosen threshold. For a detailed overview of the results, including the specific values for each metric across the three subsets, please refer to Table 2. Explainability analysis highlighted the model’s precise identification of amyloid substance, correctly marking background and nuclei areas as negative and focusing on extracellular deposits for positivity. The segmentation results indicate moderate performance, with IoU and Dice coefficients suggesting a reasonable but suboptimal overlap between predicted and ground-truth masks. The interquartile range (IQR) suggests variability across folds, indicating that segmentation accuracy may depend on specific data distributions.( Fig. 5).

**Table 2:** The table reports classification (Accuracy, F1 score, AUC-ROC) and segmentation (IoU, Dice) results across training, validation, and test sets. Values are expressed as the median with interquartile range (IQR) to capture variability across folds.

	Training	Validation	Test (tile level / WSI level)
Accuracy *median*,* IQR*	0.87 [0.87–0.88]	0.84 [0.70–0.93]	0.79 [0.73–0.84] / 0.81
F1 Score *median*,* IQR*	0.87 [0.86–0.88]	0.83 [0.67–0.90]	0.71 [0.47–0.76] / 0.86
AUC-ROC *median*,* IQR*	0.87 [0.86–0.87]	0.86 [0.80–0.90]	0.76 [0.52–0.85] / 0.78
Intersection over Union *median*,* IQR*	0.35 [0.33–0.37]	0.34 [0.28–0.46]	0.27 [0.17–0.36]
Dice Coefficient *median*,* IQR*	0.52 [0.49–0.54]	0.50 [0.43–0.62]	0.39 [0.28-0.51]

## Discussion

Increasingly recognized as a significant cause of heart failure, particularly in the elderly, CA is caused in the majority of cases by misfolded monoclonal immunoglobulin light chains from a clonal plasma cells process (AL amyloidosis) or misfolded TTR, a liver-synthesized protein that is normally involved in the transportation of the hormone thyroxine and retinol-binding protein (ATTR)^14^. The diagnostic, clinical, and therapeutic landscape of CA is rapidly evolving, with significant improvements in non-invasive detection^15,16^. Advancements in cardiac magnetic resonance (CMR) and echocardiography have significantly enhanced and serve as accurate, widely available, and sensitive diagnostic tools for assessing suspected cases^17^. Radionuclide scintigraphy complements these imaging modalities, effectively excluding alternative cardiac conditions with high sensitivity^18,19^. However, especially when used by themselves, no imaging diagnostic method reaches a perfect sensitivity and specificity, and only patients with negative monoclonal protein assessment and positive cardiac scintigraphy undergo a completely non-invasive diagnostic algorithm^20,21^. Therefore, the role of EMB is still highly valuable and is the only method to reliably establish a diagnosis in challenging cases^22^.

The digital transformation in pathology, driven by the use of scanners and virtual slides, offers numerous benefits such as improved traceability, workload efficiency, and diagnostic accuracy^23^. However, integrating amyloidosis diagnosis into this digital workflow presents challenges due to the necessity of capturing CRB for detecting amyloid deposits^9^. A possible solution to this problem is the evaluation of CRF^24^, recently validated for routine diagnosis on kidney WSI, opening the door to numerous advantages of slide digitization, such as sharing, storage and training^7^. Notably, the digital acquisition of images allowed for the first AI application for amyloid detection and segmentation and paved the way for the extension of co-registration with spatial -omics data to amyloid, as already experimented mainly in neoplastic settings^25^. Here we unprecedentedly tested an AI-based tool to infer the presence and distribution of amyloid deposits using simple H&E slides from EMB borrowing from the CRF annotations obtained with SPADA, finally undertaking the quantum leap that separates us from the real implementation of these AI instruments in the clinical setting. These findings highlight the potential for a cross-organ study of amyloidosis, which could provide insights applicable to multiple organs, while specificities of different settings (e.g. renal vs. cardiac) require approaches that may slightly differ. While the renal study focused on the challenges of differentiating renal amyloidosis from other diseases in the presence of monoclonal gammopathy of undetermined significance (MGUS), the current one emphasizes the role of EMB in complex cases where non-invasive techniques cannot definitively identify the disease. EMB, due to its invasive nature, often consists of a few and small tissue fragments, whereas renal biopsies provide larger tissue samples. Consequently, diagnostic differences also exist between these settings, such as the presence of a defined amyloid score for the kidney^26^, which is lacking for the heart, and the differing implications of disease extent, despite the direct impact of the extension of amyloid deposits on cardiac function^27^. Indeed, the inclusion of autopsy cases in this study allowed for a more comprehensive examination of amyloid deposits, offering insights that are not as easily obtained from the smaller EMB samples.

In both studies virtual slides performed comparably to traditional physical slides, aiding the digital transition in both fields. In particular, excellent interobserver agreement in identifying CA was recorded, resolving some of the historical issues associated with CR and thioflavin staining on physical slides, such as the inconsistent reproducibility of apple-green birefringence^28^. However, the CRF validation for clinical diagnosis, following College of American Pathologist guidelines, would involve using fluorescence to replace birefringence evaluation, while maintaining the rest of the diagnostic setting. This aspect will need to be addressed in future studies on a commensurate set of slides to ensure that the method can be reliably implemented in clinical practice^29^.

**Fig. 6:**
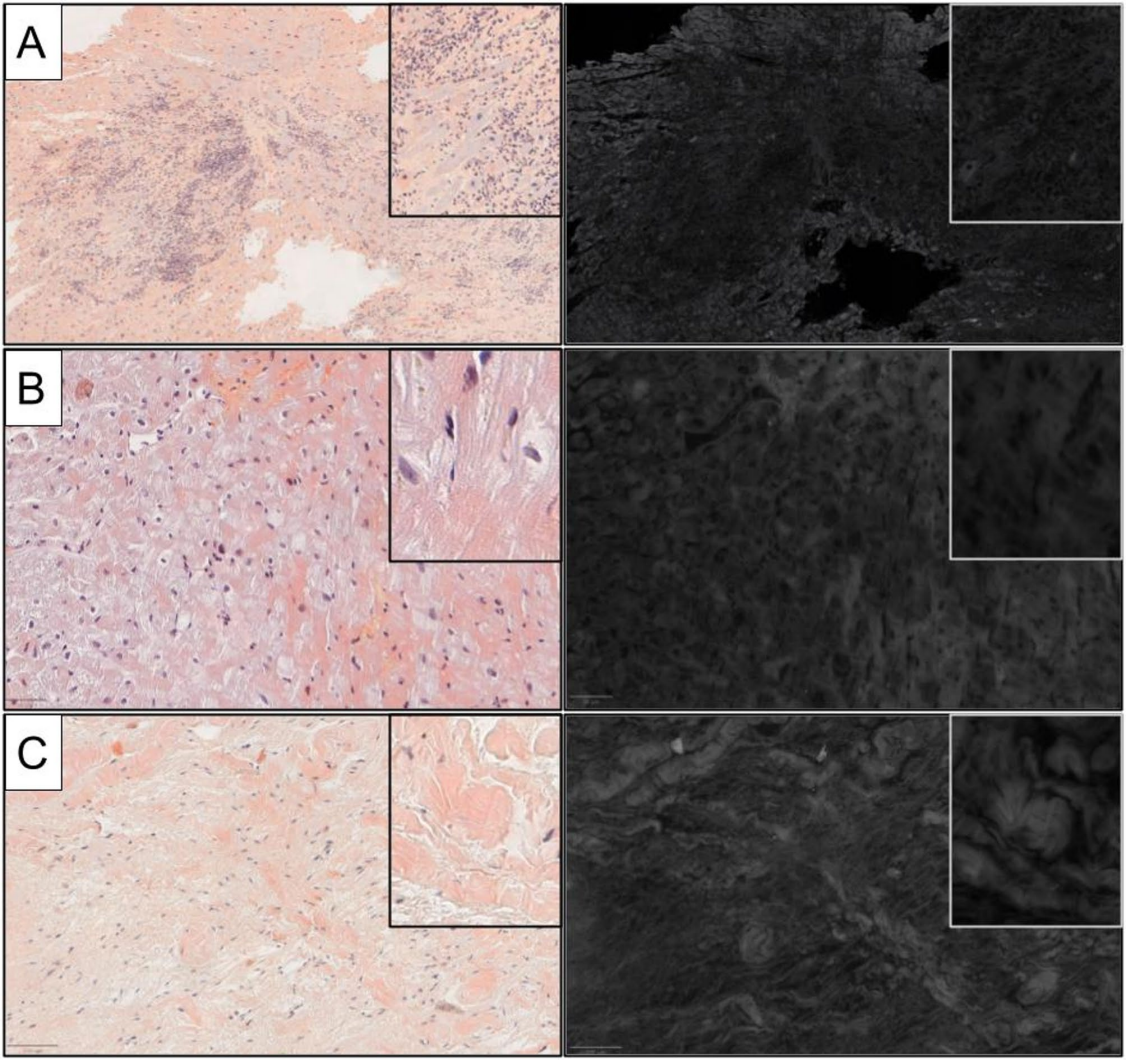
Areas of pathological cardiac tissue negative for amyloid. (**a**) Myocarditis areas with fibrosis and chronic inflammation; (**b**) and (**c**) fibrotic areas. In the virtual slides, fluorescence was negative in all cases, with minimal contrast compared to the adjacent cellular structures.

As extensively discussed, certain conditions can mimic amyloid birefringence, potentially complicating its accurate assessment. The reliability of the characteristic ‘apple green’ birefringence has also been scrutinized^30,31^. Despite Congo red, thioflavin T, and thioflavin S being very sensitive markers for amyloid, they are described as not being specific to amyloid. These findings align with the understanding that Congo red binds to many native proteins and lacks specificity for secondary structure. In our experience, both in renal pathology and in this study, false positive results from fibrotic and collagen areas are manageable when using CRF compared to CRB. Example of fibrotic area of cardiac tissue following ischemia is shown in Fig. 6^32,33^.

In cardiovascular pathology, additional stains are commonly used to detect amyloid with high accuracy^34^. Among the others, SAB, a basic dye that stains the mucopolysaccharide matrix associated with amyloid, has proven to be highly effective for screening cardiac specimens for amyloid^35^. Traditional patterns of amyloid deposition in the heart are typically classified as discrete pericellular, diffuse interstitial, or nodular^36^. Previous studies have explored correlations between these deposition patterns and amyloid types, with findings suggesting that diffuse pericellular interstitial deposits are more often associated with AL amyloidosis, while nodular deposits are more commonly linked to ATTR. Vascular involvement has also been shown to be more prevalent in AL amyloidosis^36–38^. In our study, the concordance in assessing deposition patterns did not fully align with the original diagnoses, likely due to the evaluation being based on a single staining technique and the lack of uniformity in interpreting the proposed scores. To enhance the evaluation of these secondary characteristics, introducing an additional perspective alongside the current set of slides available to pathologists could provide valuable insights. An accurate reporting of amyloid deposition patterns and vascular involvement can be beneficial for both clinical practice and research, although there is not yet a direct diagnostic role^39,40^. Indeed, direct amyloid typing—preferably through immunoelectron microscopy or liquid chromatography-tandem mass spectrometry (LC-MS/MS)—remains the cornerstone for diagnosing and treating CA^41^. From a practical standpoint, CRF, due to its high sensitivity as demonstrated by Linke et al., has shown remarkable sensitivity even for small deposits. However, maintaining specificity—especially in complex or ambiguous cases—may still require coupling it with CRB or other staining techniques^33^.

The SPADA pipeline served multiple purposes in this study. In the single case of discrepancy, the computational approach provided a refined view of the suspicious area by subtracting tissue autofluorescence, which enhanced visualization of minimal vascular deposition and enabled consensus between the two observers, aligning with the final diagnosis. However, in this case, traditional methods also revealed amyloid deposits at the pericellular level. Similarly, despite a strong positive correlation between SPADA morphometry at CRF and traditional staining techniques (such as Hematoxylin-Eosin and SAB), some discordance was noted. This inconsistency is likely due to fading artifacts that gradually diminish color intensity in scanned images. Specific differences compared to SAB staining have not been noted, as demonstrated by the high concordance between the evaluations. However, particularly in surgical samples, there appears to be a generally smaller proportion of deposits identified using CRF. Addressing this issue by optimizing fluorescence scanning parameters, particularly for larger histological sections, could improve analysis accuracy and reduce discrepancies in future studies.

The SPADA pipeline also allowed the development of an AI tool designed for centers lacking fluorescence scanners equipped with a Texas red filter. By training the model on automatically annotated virtual slides and validating its performance, this study pioneers the integration of AI capabilities into the detection and classification of amyloidosis in pathology, providing a ground truth that would otherwise be challenging to scale up and would not allow for the creation of a sufficiently large dataset for training purposes. This innovative approach helps pathologists by allowing them to scan entire sets of slides, enabling the detection of minimal or focal deposits^42^. Recent developments are increasingly highlighting the integration of AI in this field, offering exciting possibilities for innovation. A recent study by Mukherjee et al. trained two models to first identify amyloid deposits based on specific infrared spectral peaks, and then to classify these deposits as either AL or ATTR^12^. Another advancement involved a deep learning approach that enables label-free detection of amyloid deposits by simulating birefringence imaging and CR staining through tissue autofluorescence textures. This method generates virtual images that mimic traditional CR-stained tissue in both brightfield and polarized light, allowing for accurate identification of amyloid deposits without the need for physical staining. Collectively, these developments mark a transformative step forward in the diagnosis and management of amyloidosis, making second-level methods accessible to unequipped laboratories through the use of AI, potentially serving as a screening tool to flag positive cases^43^.

### Limitations and future directions

While the study presents promising results, certain limitations should be acknowledged. The sample size is relatively small, but could be extended by including diverse cardiovascular specimens (e.g., apical cores and atrial appendages). Additionally, the study primarily focused on the technical aspects of CRF and AI integration, with limited exploration of clinical data. Future research should aim to expand the sample size, to include a broader range of clinical scenarios onlarger, multicentric cohorts to ensure its reproducibility across different settings. Another key aspect to address is testing these methods on selected cases with histologically challenging amyloidosis manifestations and greater biological variability, to assess their robustness in complex diagnostic scenarios.

## Conclusion

The study emphasizes the transformative impact of CRF on virtual slides and the integration of AI in diagnosing CA. The combination of CRF’s high reliability and diagnostic accuracy with AI’s efficiency represents a significant advancement in digital pathology, potentially streamlining the routine diagnostic process for CA and aiding in the segmentation of deposits for future -omics analyses. Promising results were achieved in tile-based classification, notably with a high negative predictive value. As the training cohort grows and further validations are performed, this approach could enhance histological case screening by guiding pathologists to areas needing closer examination for amyloidosis.

## Electronic supplementary material

Below is the link to the electronic supplementary material.

Supplementary Material 1

## Data Availability

Spreadsheets and codes are deposited in a GitHub repository available at https://github.com/Gizmopath/Cardiac-Amyloid. The full ImageJ macro script is available at https://github.com/Gizmopath/Amyloid.
