# Supplementary material for: Congo red fluorescence enhances digital pathology workflow in cardiac amyloidosis
Source: Sci Rep. 2025 Jul 11;15:25089. doi: 10.1038/s41598-025-07157-5 (PMC12254226; doi:10.1038/s41598-025-07157-5)

**Supplementary Figure 1.** Concordance between observers: confusion matrices show the agreement of Congo Red fluorescence (CRF) assessment with the original diagnosis (left) and CRF inter-observer agreement (right).


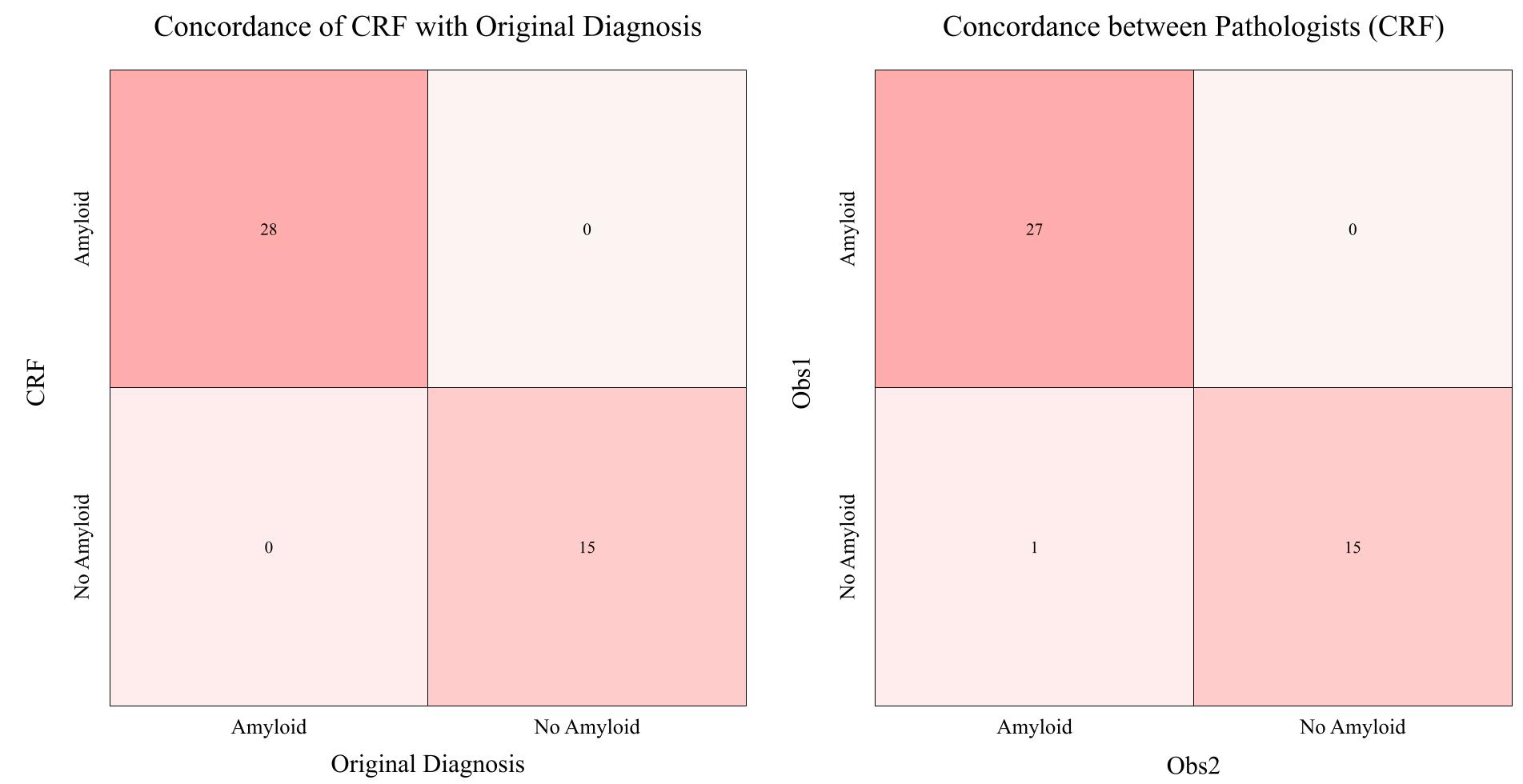

Supplement: Supplementary file 1 — Supplementary Material 1 [file 41598_2025_7157_MOESM1_ESM.docx]
